# Enhancing the capabilities of forcibly displaced people: a human development approach to conflict- and displacement-related stressors

**DOI:** 10.1017/S2045796021000263

**Published:** 2021-04-29

**Authors:** Ross G. White, Catharina Van der Boor

**Affiliations:** Institute of Population Health, University of Liverpool, Brownlow Hill, Liverpool, L69 3BX, UK

**Keywords:** Mental health, other psychosocial techniques/treatments, psychotherapy, social environment

## Abstract

**Aims:**

The mental health of individuals who have been forcibly displaced can be impacted both by war-related traumatic events and *displacement-related stressors*, which arise as a consequence of their migratory journey and subsequent experiences. In addition to focusing on mental disorders, there is a need to explore broader psychosocial outcomes that are important for forcibly displaced people. Our aim is to present a coherent explanatory framework to understand *how* both past traumatic events and ongoing stressors operating throughout forcibly displaced people's social environment can impact mental health and psychosocial wellbeing.

**Methods:**

We describe the *capability approach* (CA), a human development framework that foregrounds individuals’ freedom to engage in forms of being and doing that are valuable to them. We consider the opportunities that the CA provides for understanding how a myriad of factors can impact forcibly displaced people, and how different forms of support can be configured to meet the needs of particular people and communities.

**Results:**

The CA recognises that various factors can share a common putative causal mechanism in their impact on forcibly displaced people, i.e. these factors limit a person's ability to develop capabilities and their freedom to engage in valued forms of being and doing. The rights based ethos of the CA enables multisectoral and coordinated activity, which can be directed towards addressing factors across the social environment. Importantly, the CA helps to explain why particular forms of support may be more beneficial for individuals or communities at certain times compared to others.

**Conclusion:**

The application of the CA can help to guard against the risk that the aspirations of assessment instruments and interventions aimed at supporting forcibly displaced people are narrowly focused on addressing distress and disorders, to instead adopt a more expansive focus on forcibly displaced people's potential and the possibilities that they wish to realise.

The number of forcibly displaced people (FDP) is at an all-time high of 80 million, equating to 1% of all humanity (UNHCR, 2020). The majority of these individuals (46 million) have been internally displaced, with the remainder being forced to leave their country of origin as refugees or asylum seekers (UNHCR, 2020). It is widely recognised that forced displacement can impact people's mental health. A recent meta-analysis of studies conducted in low- or middle-income countries with people who had been internally displaced or who had fled to neighbouring countries due to conflict, estimated that 22% of people had experienced mental disorders (depression, anxiety disorder, post-traumatic stress disorder (PTSD), bipolar disorder, and/or schizophrenia) and that 9% had experienced a moderate to severe form of mental disorder (Charlson *et al*., 2019). A meta-analysis of studies conducted in high-income countries that recruited refugees or asylum seekers found prevalence rates of 13% for anxiety disorder, 30% for depression, and 29% for PTSD (Henkelmann *et al*., 2020).

## War-related *v.* displacement-related stressors

On proposing the *Ecological Model of Refugee Distress*, Miller and Rasmussen (2017) highlighted that, in addition to war-related traumatic events, the mental health of FDP can also be impacted by *displacement-related stressors* (e.g. uncertainty regarding asylum status, loss of social support networks, relationship difficulties, etc.). Four specific factors were highlighted by Miller and Rasmussen (2010*a*, 2010*b*) to be contributing to the association between displacement-related stressors and mental-health difficulties, namely: temporal proximity of the stress, lack of control over the stressors, pervasiveness of the stress and wide-range of potential stressors. Although Miller and Rasmussen’s (2017) model provides valuable insights into the question: ‘why are displacement-related stressors so strongly linked to mental health?’ (p. 6), there remains a need for coherent explanatory frameworks aimed at understanding *how* both conflict-related trauma and ongoing stressors operating across different strata of the displaced person's social environment contribute to difficulties with mental health and wellbeing. Such frameworks will help to address the need for greater conceptual clarity regarding efforts being made to support FDP (Miller *et al*., 2021). These frameworks will need to be flexible enough to account for both similarities and important differences in the experiences of different types of FDP (internally displaced people, refugees and asylum seekers) and the contexts that they exist in.

## Mental health, psychosocial wellbeing and human development

Although research evidence indicates that varying proportions of FDP meet the criteria for mental disorders, it is important to note that a considerable proportion does not. To more fully understand the experience of FDP, there is a need to broaden the focus beyond psychopathology to other outcomes relevant to psychosocial functioning, which relate to the interaction of the individual and their environment and inter-personal context. Mental health has after-all been defined as ‘a state of wellbeing in which every individual realizes his or her own potential, can cope with the normal stresses of life, can work productively and fruitfully, and is able to make a contribution to her or his community’ (WHO, 2013). Focusing on wellbeing allows a shift from a narrow focus on the presence or absence of mental illness alone to a fuller, richer consideration of what factors can bring vitality to the person's lived experiences. Unfortunately, there has been a comparative lack of research focusing on subjective wellbeing and quality of life of FDP (Turrini *et al*., 2019; van der Boor *et al*., 2020*a*). We believe that, rather than being viewed exclusively as a *health* issue, the mental health and psychosocial wellbeing of FDP needs to be understood as a *human rights* issue that includes a focus on key principles such as participation in society, non-discrimination, human dignity and empowerment.

In recent decades, the *Adaptation and Development After Persecution and Trauma* (ADAPT; Silove *et al*., 2006; Silove, 2013) model has been proposed as a conceptual framework for providing mental health and psychosocial support (MHPSS) in post-conflict situations. The framework, which was developed as a consequence of efforts that were made to support conflict-affected populations in East Timor, details five core ‘pillars’ that are purported to be disrupted by forced displacement and/or conflict: (1) safety/security; (2) bonds/networks; (3) justice; (4) roles and identities and (5) existential meaning. The framework was intended as a heuristic to assist those working to support conflict-affected populations. Those who developed the ADAPT model recognised the potential for it to be further developed or replaced with a more comprehensive model as knowledge developed (Silove, 2013).

## The capability approach

We contend that the *capability approach* (CA; Sen, 1999, Nussbaum, 2000), a human development approach that foregrounds individuals’ freedom to engage in forms of being and doing (or *functionings*) that are valuable to them, provides scope to identify factors impacting on the mental health and wellbeing of FDP. From a CA perspective, ‘freedom’ is purported to have two important aspects: (1) agency (i.e. the ability of an individual to act on behalf of what matters to them and (2) capability (i.e. the potential to achieve valuable functionings from various good opportunities).

Nussbaum (2000) proposed a list of ten central capabilities that she claimed essential to sustain human life and dignity, namely: life; bodily health; bodily integrity; senses, imagination and thought; emotion; practical reason; affiliation; other species; play and control over one's environment. However, rather than being prescriptive about which capabilities should be prioritised, Sen (2004, p. 45) highlights a need to work with communities collaboratively to identify inductively what is valued by people in different contexts. Sen posits that although identifying needs is clearly important, it is crucial to understand what the satisfaction of those particular needs can afford for those individuals in terms of what truly matters to them. Whereas the satisfaction of needs might on occasion be perfunctory, the enhancement of capabilities is characterised by a sense of vitality. An important aspect of the CA is the focus it allocates to what are referred to as *conversion factors*; the context-specific circumstances that allow people to convert available resources into capabilities. Conversion factors can exist at various different levels of scale in the person's social environment (i.e. the individual, interpersonal, community and institutional system levels) and can include factors such as knowledge acquisition, social support, access to services, government policy, etc. The following description by Robeyns (2005) provides a helpful overview of the CA as follows:

According to the capability approach, the ends of wellbeing, justice and development should be conceptualized in terms of people's capabilities to function; that is, their effective opportunities to undertake the actions and activities that they want to engage in and be whom they want to be…The distinction between achieved functionings and capabilities is between the realized and the effectively possible (p. 95).

A number of recent studies have found that CA-derived outcomes are strongly associated with mental health and social outcomes in adult populations living in high-income countries (Brunner, 2017; Mitchell *et al*., 2017; Vergunst *et al*., 2017; Hackert *et al*., 2019).

### Applying the CA to the experience of FDP

We propose that the CA framework provides good scope for inductively exploring the breadth of factors (including war-related trauma and loss, displacement-related stressors and other forms of daily stress) that can impact the mental health and wellbeing of FDP. The CA does so by recognising that these diverse factors share a common putative causal mechanism, i.e. they limit a person's freedom to engage in forms of being and doing which they value. Emerging research evidence has applied the CA to the experience of FDP. Chase (2020) conducted ethnographic and qualitative research in the United Kingdom with 31 unaccompanied young people (17–25 years of age) from Afghanistan to explore their experience of transitioning into adulthood from a CA perspective. Eleven of the participants had been forcibly returned to Afghanistan at some point, with ten remigrating to the United Kingdom, Indonesia, Germany, Italy, Bulgaria or Pakistan at the time of the interview. Key capabilities that emerged as being important were: (1) safety, freedom and choice to build and realise a ‘better future’; (2) notions of identity and belonging; (3) constructing viable futures that extend beyond the constraints of an institutional focus on their migratory status; (4) maintaining bodily strength, and mental and emotional wellbeing and (5) freedom to build and sustain relationships with others. Chase (2020) concluded that the application of the CA highlighted a need to ‘move beyond the provision of basic needs and protection of children and a critical assessment of the freedoms they require to enter adulthood, take command of their environments and create the futures they aspire to’ (p. 453).

Using a CA-informed approach, we conducted focus group discussions with 16 female refugees living in the United Kingdom (van der Boor *et al*., 2020*b*). The participants all had temporary leave to remain in the United Kingdom (i.e. permission to remain for up to 5 years during which time an application to have indefinite leave to remain can be considered) and were able to converse comfortably in English. Participants came from a variety of different countries including Azerbaijan, Sudan/South Sudan, Sierra Leone, Nigeria, Iran and Syria. The discussions started with an open-ended exploration of the meaning of a ‘good life’ for refugees (What does the term good life mean to you?). An interpretative phenomenological analysis was used to identify key issues/themes. Three main themes were identified, namely: *legal security* (i.e. feeling protected by the law), *personal agency* (i.e. being able to control one's own thoughts, feelings and actions) and *social cohesion* (i.e. feeling connected to other members of society) (van der Boor *et al*., 2020*b*). Similarly, in a recent study, we used one-to-one semi-structured interviews to engage with 60 adult male and female Congolese refugees living in two refugee settlements in Uganda and Rwanda to investigate what a ‘good life’ meant to them (What does ‘having a good life’ mean to you?) (Robinson *et al*., 2021). Supplementary to having basic needs relating to food and shelter met, both male and female participants identified being well dressed and being clean to be important to achieve a ‘good life.’ This was associated with gaining the respect of other people and maintaining good relationships/avoiding conflict with neighbours. Some gender-specific distinctions were also noted; women's aspirations focused on the wellbeing of their children and the material fabric of their homes, and men foregrounded opportunities for employment, material possessions that demonstrate their status, and opportunities for greater public participation in community life. The findings of these two studies highlight that different capabilities are relevant to different contexts, and can be influenced by personal, interpersonal and institutional factors. Furthermore, similar to the study conducted by Chase (2020), these findings indicate the need to move beyond a focus on basic needs to specifically assess the real opportunities and freedoms FDP have to live a life that is valuable to them, and to resource and configure forms of support offered to them accordingly.

**Fig. 1.**
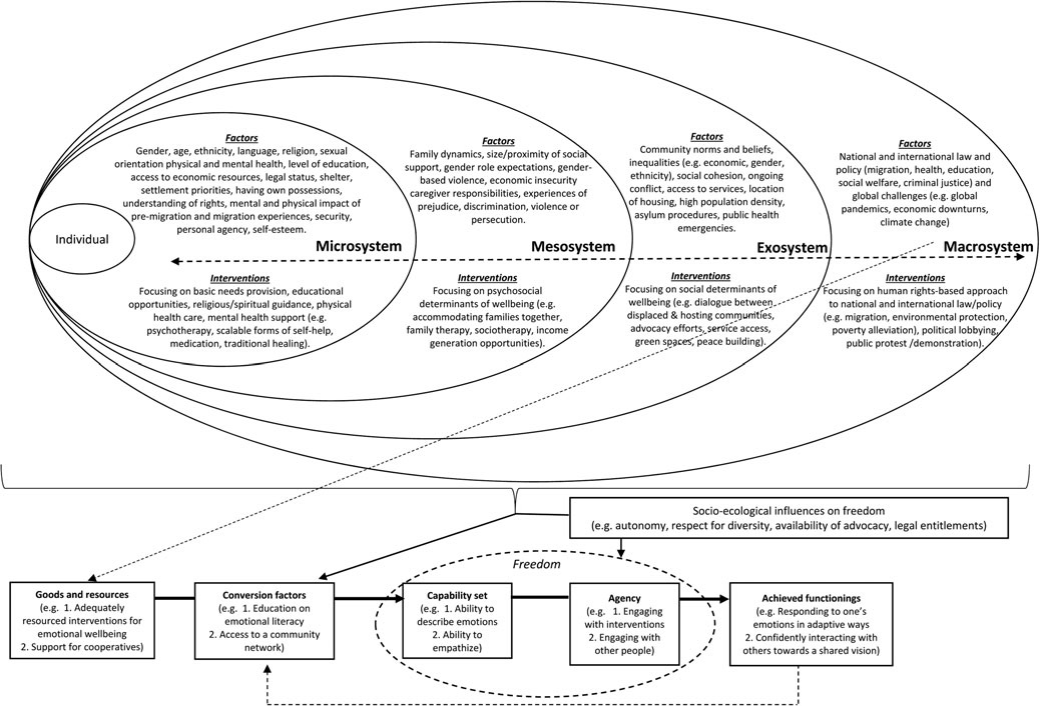
A capability approach for understanding and supporting the wellbeing of forcibly displaced people.

The CA helps to elucidate a focus on what living well means to FDP, what community resources are available, and how these resources interact with the individuals’ capabilities and freedom to engage in valuable functionings. Figure 1 provides a diagrammatic representation of a capability-informed approach to both understanding and supporting the wellbeing of FDP. In recognition of the ways in which factors operate at different levels of scale (Trani *et al*., 2011), it includes a specific focus on factors across the following strata of FDP's social environment:

(1) The *microsystem* (i.e. factors impacting directly on the individual such as age, sex, talent, impairment etc.).
(2) The *mesosystem* (i.e. factors directly impacting on the social experience of the individual such as family support, care-giving responsibilities, domestic abuse etc.).
(3) The *exosystem* (i.e. factors that are not directly experienced by the individual but experienced by those in the person's social networks/community, e.g. the impact of media portrayals of FDP on local levels of discrimination).
(4) The *macrosystem* (i.e. factors that operate at an institutional level including laws and policies).

Figure 1 also highlights how the CA can guide the coordination of interventions operating across the different levels of the forcibly displaced person's social environment (from the *microsystem* to the *macrosystem*) to enhance capabilities, promote agency and support valued functionings, which can in turn act as a catalyst for further capability development. According to a capability-informed approach for supporting the mental health and wellbeing of FDP, the character and focus of interventions will vary across the levels from the clinical (*microsystem*, e.g. one-to-one psychotherapy), to the psychosocial (*mesosystem*, e.g. forms of sociotherapy), to the social (*exosystem* e.g. peace-building programmes), to the societal *(macrosystem*, e.g. legislative change).

It is important to recognise that actions of the State at the macrosystem level can impact the experience of FDP across the other levels of the social environment. These macrosystem factors include (but are not limited to) government policy on migration, welfare, health, education, employment and criminal justice. The attritional nature of the structures and systems that FDP face has been described by de Vries and Guild (2019) as the ‘politics of exhaustion’ (p. 2156). These impacts can be felt at the various levels of the person's social environment in that the implementation of policy, or lack thereof, has implications for the communities in which the person lives, their social networks, and the individual themselves. As presented in Fig. 1, the CA provides a lens to scrutinise *how* these impacts manifest in the person's experience in relation to the availability of goods and resources, conversion factors, capabilities, agency and/or functionings. The CA does not presume to provide easy solutions to the predicaments and dilemmas that FDP experience, rather it serves to emphasise the need for careful consideration of opportunities and barriers operating at different levels of the person's social environment so that support efforts can be tailored to the person in the context in which they exist. This will help to identify where the opportunities and responsibility for change reside – with the individual, community and/or the macrosystem structures and institutions.

The CA provides scope for recognising and embracing the contribution of purportedly alternative explanatory frameworks (e.g. *clinical v*. *social-environmental*), which are proposed for understanding the experience of FDP (Miller *et al*., 2021). The application of the CA to the experiences of FDP is consistent with the ethos of the MHPSS approach as well as influential guidelines relating to humanitarian crises such as the *mhGAP Humanitarian Intervention Guide: Clinical management of mental, neurological and substance use conditions in humanitarian emergencies* (WHO and UNHCR, 2015) which details important principles and processes for providing support, the *Sphere Handbook* (Sphere Association, 2018) which identified the need to ‘Coordinate mental health and psychosocial supports across sectors’ (p. 399), and the IASC (2007) *Guidelines on mental Health and Psychosocial Support in Emergency Settings* which aim ‘to enable humanitarian actors to plan, establish and coordinate a set of minimum multi-sectoral responses to protect and improve people's mental health and psychosocial wellbeing in the midst of an emergency’ (p. iii). The application of the CA to the experiences of FDP is also broadly consistent with Tol’s (2020) recent call for the application of a social justice framework for guiding research and practice relating to the association between interpersonal violence and mental health – albeit that call was not focused specifically on FDP.

### A CA perspective on human rights

The CA has made a valuable contribution to efforts aimed at further operationalising concepts such as social justice and human rights. Specifically, proponents of the CA (e.g. Nussbaum, 2002; Vizard 2007; Burchardt and Vizard 2011) have highlighted a need to move beyond a tendency to express human rights in terms of ‘negative freedoms’ (i.e. the prevention of harm) to instead articulate these more fully as ‘positive freedoms’ (i.e. the freedom to be and do what one values). Burchardt and Vizard (2011) described a two-stage procedure by which the ‘bottom up’ generation of capability lists by local communities was balanced against internationally recognised human rights principles and standards (specifically *The International Covenant on Civil and Political Rights* (UN, 1966*a*) and *The International Covenant on Economic, Social and Cultural Rights* (UN, 1966*b*)) to generate a capability set that contributed to the development of the *Equality and Human Rights Commission (United Kingdom) Measurement Framework* (EHRC, 2017) to assess equality and human rights provision in the United Kingdom. Furthermore, by highlighting *agency* as an interactional process in which power struggles can influence what constitutes ‘genuine choices’ for a person, the CA has helped to refine the understanding of the social dynamics that influence fairness and justice (Wolff and de-Shalit, 2007).

Figure 1 illustrates the reciprocal relationships that can exist between human rights and mental health and psychosocial functioning across the social environment. The absence of human rights legislation (macrosystem level) and/or a lack of implementation of protection principles in communities (exosystem level) may permit abusive or neglectful interactions (mesosystem level), which can negatively impact a forcibly displaced person's wellbeing (microsystem level). Equally, even when human rights protections are available and implemented, the ability of FDP to avail of these protections may be hindered by factors operating at the microsystem level, such as demoralisation, feelings of anxiety and/or worries about life circumstances and future prospects (Harrison *et al*., 2021) that can inhibit their capabilities and functionings.

## Implications for intervention

A recent meta-analysis exploring the effectiveness and acceptability of psychological interventions for refugees and asylum seekers noted that psychological interventions have a significant effect on PTSD, depression and anxiety outcomes (Turrini *et al*., 2019). However, the authors caution that the evidence is of moderate quality and studies have tended to recruit comparatively small sample sizes. In a move away from a focus on disorder-specific outcomes, Tol *et al*. (2020) recently conducted a large randomised controlled trial (*n* = 694) in northern Uganda that found that the five-session group-based *Self-Help Plus* (SH + ) intervention developed by the *World Health Organization* (WHO) reduced levels of distress (Cohen's *d* = –0.26; assessed by the K-6 scale; Kessler *et al*., 2003) and increased wellbeing (Cohen's *d* = 0.36; assessed by the WHO-5; WHO, 1998) three months after baseline assessments. SH + is based on acceptance and commitment therapy (ACT; Hayes *et al*., 1999). Like the CA, ACT places specific emphasis on exploring what people value and supporting them to commit to actions that are consistent with their values.

We believe that the CA has a number of important implications for interventions aimed at supporting the mental health and wellbeing of forcibly displaced populations. First, the CA recognises that the interventions a person requires will depend on the opportunities and barriers that they are facing in efforts to enhance their capabilities. Second, the rights based, human-development ethos of the CA highlights the need for multisectoral and coordinated activity to address factors across the different strata of the social environment that enhance or restrict opportunities for people to engage in valued functionings. Third, the CA highlights that the sequencing of interventions needs to be carefully considered. For example, a person's ability to benefit from a community-based psychosocial intervention may first require them to develop their literacy level, and a person engaging with a microcredit programme may require support with managing the difficult thoughts and emotions that are impacting on their concentration. Finally, the importance that the CA allocates to people's agency emphasises the importance of interventions working to empower FDP to choose what capabilities and functionings are most important to them. Research has found that agency is a key issue for FDP (Korac, 2009; van der Boor *et al*., 2020*b*).

As such, the CA helps us to understand why particular types of interventions may be helpful for some FDP but not others, and why certain forms of intervention may be necessary but not sufficient to meaningfully improve mental health and wellbeing. For example, whilst the evidence seems to suggest that psychological interventions can bring about positive change for a significant proportion of people, it may be that these interventions need to be delivered in coordination with other forms of support including (but not restricted to): positive social interactions, poverty alleviation programmes (e.g. microcredit schemes), educational opportunities, access to transportation, affordance of legal protections (e.g. leave to remain in the host country), etc. Although the idea that particular interventions will be uniformly effective for enhancing the wellbeing of large groups of people has an understandable appeal, it is clear that people are not all uniform and support has to be tailored to the needs of the individual and the social environment that they live in.

The *Inter-Agency Standing Committee* (IASC, 2019) have articulated the need to ‘treat MHPSS as a cross-cutting issue that has relevance within health, protection, nutrition, education and CCCM sectors/clusters, in all emergencies….(and provide) Support for the creation of, and the work of, country-level MHPSS Working Groups in all migration, refugee and humanitarian contexts as crosscutting groups’ (p. 5). We wholeheartedly agree that multi-sectoral, cross-cutting work that speaks directly to MHPSS is required. However, in the absence of a guiding person-centred CA-informed formulation, these efforts may struggle to have the desired impact. In particular, there is a need to better understand the gaps that exist between peoples’ capabilities (i.e. what the person has the potential to do) and their actual functioning (i.e. the forms of valued functionings that the individual is able to perform) (Trani *et al*., 2011). Moving forward, the following CA-informed questions may prove helpful for guiding policy, resource allocation and support provision:

(1) What efforts are being made to understand what a good life means to the FDP and their communities?
(2) What conversion factors operating at different strata of the social environment might facilitate or restrict capability enhancement of FDP?
(3) How can multi-sectoral support and interventions be configured to enhance a forcibly displaced person's capabilities and enable their valued functionings?
(4) How can the agency of FDP be appropriately fostered in the social environment in which they live?

Building on Fig. 1, we propose a *CA to Formulating Experiences* (CAFE) tool to illustrate how the CA can be used to enrich understanding of factors from across the individuals’ social environment that may be detrimental or protective for mental health and wellbeing and to help guide efforts to configure support. This tool draws on the process and principles of psychological formulation that builds a shared understanding of the nature of the problems being experienced, as well as relevant predisposing (background), precipitating (triggering), perpetuating (maintaining) and protective (resilience) factors. We propose that crucial pre-requisites for the completion of a CAFE are (1) a detailed situational analysis of relevant factors pertaining to the social environment of the individual; (2) inductive qualitative work aimed at identifying capability sets that FDP living in that context consider being important. The predefined capability sets can be used to guide the discussions between the humanitarian worker and the forcibly displaced person when the CAFE tool is being completed – with particular attention being drawn to the capabilities that the displaced individual wishes to prioritise; (3) good working knowledge of the local and national governance structures (including relevant policies and legislation pertaining to FDP).

**Fig. 2.**
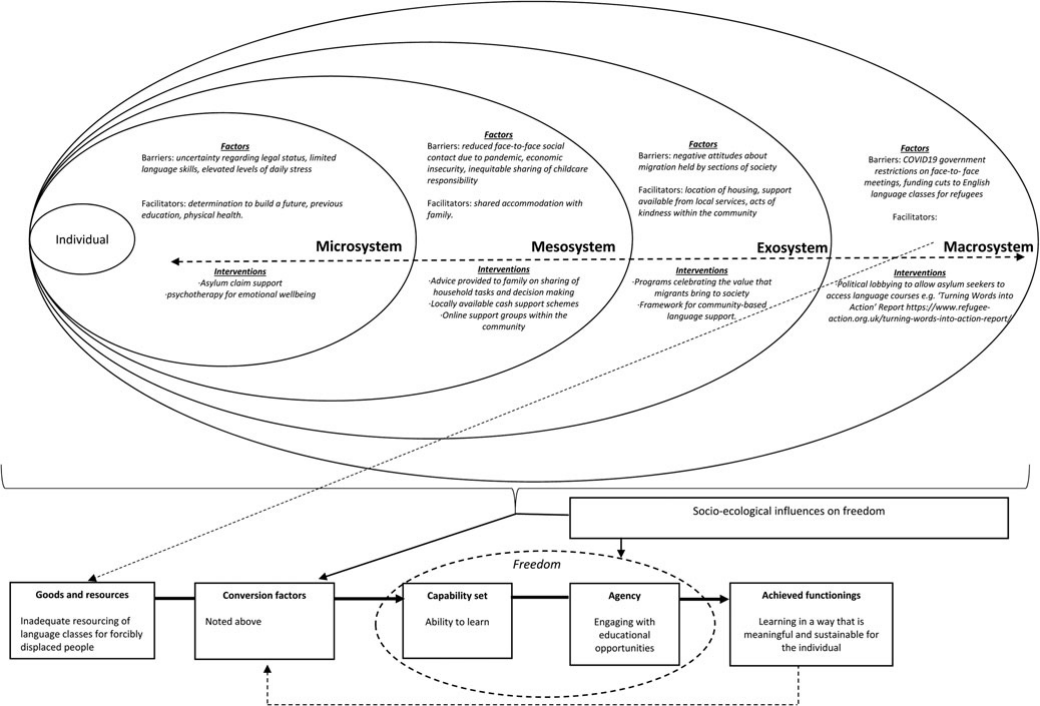
A Capability Approach Formulation of the Experiences (CAFE) of a 30-year-old woman who has migrated to the United Kingdom with her husband and young children to seek asylum. This example explores the domain of building a future through accessing education, previously identified as important for women seeking refuge in the United Kingdom (van der Boor et al., 2020*b*).

**Fig. 3.**
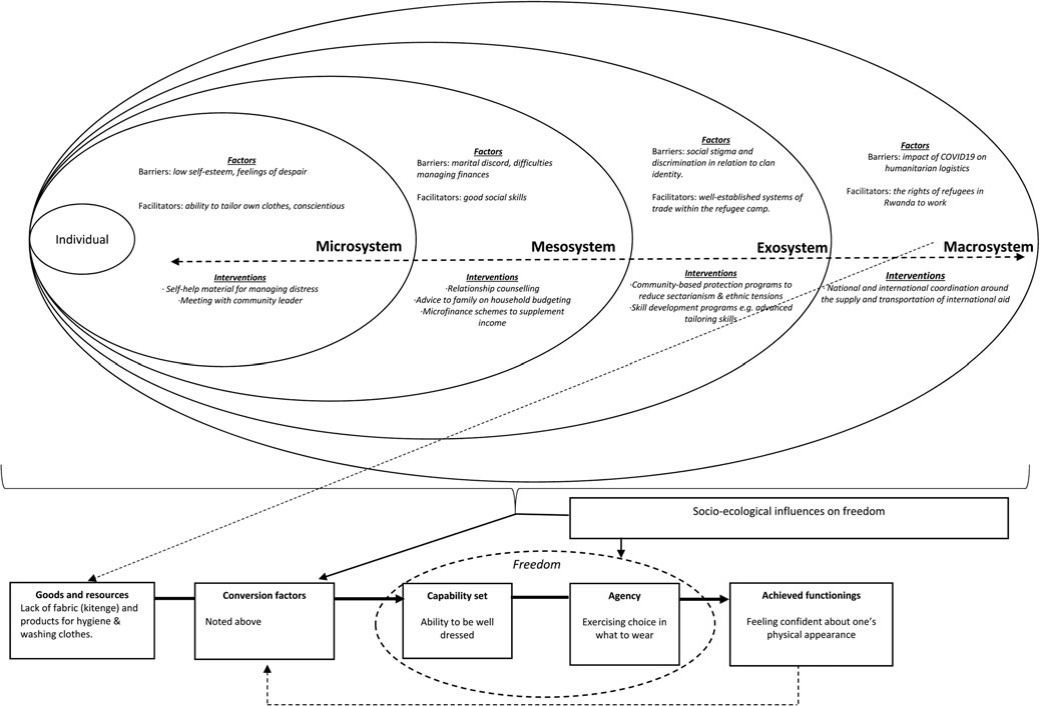
A Capability Approach Formulation of the Experiences (CAFE) of a 25-year-old man residing in a refugee community in Rwanda. This example explores the domain of being well dressed, previously identified as important for Congolese refugees residing in Rwanda and Uganda (Robinson et al., 2021).

The CAFE can then be used to identify interventions at the appropriate levels of the social environment, allowing support provision to be tailored to the individual's needs. Two hypothetical examples drawing on capabilities identified during our research with female refugees in the United Kingdom (see Fig. 2), and with Congolese refugees in Rwanda and Uganda (see Fig. 3) are provided. These examples are intended to highlight how various forms of intervention operating across different sectors (e.g. education, health and community protection), with distinct objectives (e.g. managing stress, improving community attitudes to migrants and protecting human rights) and separate modes of delivery (e.g. self-help, community groups and public campaigns), can be configured with the unifying aim of supporting the forcibly displaced person to enhance particular capabilities and perform associated valued functionings.

The CAFE tool may prove helpful for staff working with organisations/agencies to determine what forms of support should be prioritised, and to understand what broader contextual factors may serve to facilitate or thwart these efforts. The completion of the CAFE may also be valuable for informing discussions at multi-agency meetings such as the *MHPSS Working Groups* that are active in humanitarian settings to discuss the configuration and coordination of support programmes. There are over 50 of these currently active across the globe (Harrison *et al*., 2021). In particular, the CAFE could support multi-agency forums identify: (1) potential interventions that may not be currently available (due to financial and logistical constraints, or a lack of sufficient expertise) and (2) factors influencing mental health and wellbeing that cannot be sufficiently addressed at the local level alone (i.e. forms of structural violence operating at the exo- and macro-system levels, e.g. discrimination). Coordinated efforts by organisations and multi-agency groups will be necessary to bring about the requisite policy, legislative and budgetary changes that can prove crucial for enhancing the capabilities and valued functionings of FDP. If the structural barriers which can limit the freedom of FDP to enhance their capabilities remain unaddressed, then interventions operating at other levels of the social environment to support FDP will have limited effectiveness.

## Conclusion

The application of the CA provides a flexible framework for understanding how a myriad of factors (including war-related trauma and displacement-related stressors) might affect FDP, whilst simultaneously helping to broaden the focus of interventions from narrowly targeting distress and disorders to focusing more on people's potential and the possibilities that they wish to realise.

## Availability of data and materials

This is a conceptual paper, and no primary data was used in guiding the creation of the manuscript. Consequently, there is no data or associated material to be shared.
